# A flow cytometric assay to quantify invasion of red blood cells by rodent *Plasmodium* parasites *in vivo*

**DOI:** 10.1186/1475-2875-13-100

**Published:** 2014-03-17

**Authors:** Patrick M Lelliott, Shelley Lampkin, Brendan J McMorran, Simon J Foote, Gaetan Burgio

**Affiliations:** 1 Australian School of Advanced Medicine, Macquarie University, Sydney, NSW, Australia

**Keywords:** Malaria, Plasmodium chabaudi, Plasmodium berghei, Flow cytometry, Parasitaemia, Merozoite, Invasion, *In vivo*. JC-1

## Abstract

**Background:**

Malaria treatments are becoming less effective due to the rapid spread of drug resistant parasites. Increased understanding of the host/parasite interaction is crucial in order to develop treatments that will be less prone to resistance. Parasite invasion of the red blood cell (RBC) is a critical aspect of the parasite life cycle and is, therefore, a promising target for the development of malaria treatments. Assays for analysing parasite invasion *in vitro* have been developed, but no equivalent assays exist for *in vivo* studies. This article describes a novel flow cytometric *in vivo* parasite invasion assay.

**Methods:**

Experiments were conducted with mice infected with erythrocytic stages of *Plasmodium chabaudi adami* strain DS. Exogenously labelled blood cells were transfused into infected mice at schizogony, and collected blood samples stained and analysed using flow cytometry to specifically detect and measure proportions of labelled RBC containing newly invaded parasites. A combination of antibodies (CD45 and CD71) and fluorescent dyes, Hoechst (DNA) and JC-1 (mitochondrial membrane potential), were used to differentiate parasitized RBCs from uninfected cells, RBCs containing Howell-Jolly bodies, leukocytes and RBC progenitors. Blood cells were treated *ex vivo* with proteases to examine the effects on *in vivo* parasite invasion.

**Results:**

The staining and flow cytometry analysis method was accurate in determining the parasitaemia down to 0.013% with the limit of detection at 0.007%. Transfused labelled blood supported normal rates of parasite invasion. Protease-treated red cells resulted in 35% decrease in the rate of parasite invasion within 30 minutes of introduction into the bloodstream of infected mice.

**Conclusions:**

The invasion assay presented here is a versatile method for the study of *in vivo* red cell invasion efficiency of Plasmodium parasites in mice, and allows direct comparison of invasion in red cells derived from two different populations. The method also serves as an accurate alternative method of estimating blood parasitaemia.

## Background

Malaria is one of the most deadly infectious diseases, resulting in nearly one million deaths annually [1]. The symptomatic stage of infection occurs when the merozoite form of the Plasmodium parasite invades circulating red blood cells (RBCs), and undergoes development and replication. Interfering with merozoite invasion is regarded a potentially useful and novel anti-malarial approach, and understanding of the process is relatively advanced. The merozoite first binds to the RBC at an indiscriminate orientation before aligning itself so its apical end faces the RBC surface whereby its binding becomes irreversible [2]. Parasite proteins are then secreted resulting in remodelling of the RBC surface, tight junction formation, and endocytosis of the merozoite [3-6]. Genetic variations in the parasite [7-11] and the host [12,13] have been reported to alter parasite invasion efficiency, and several key protein interactions have been identified in this process [4,14-16]. However, all of these studies have been conducted using *in vitro* cultured parasites [5,8,17-19], and there is a need for methods to test and translate these findings *in vivo*. This is of particular importance when considering the interaction between the host’s immune system and invading merozoite. Indeed, during infection the production of antibodies against merozoite antigens, which inhibit invasion, is thought to be an important mechanism involved in malaria immunity [20-22]. In concordance with this, it has been shown that the parasite possesses several alternate invasion pathways, and that it may switch between these pathways in response to immune action [5,7,23-25]. The development of vaccines against merozoite antigens, or other invasion blocking therapies, may, therefore, benefit from an *in vivo* invasion assessment, which would account for the role of the immune system in this interaction.

Rodent malaria parasites have long been utilized as a model for human malaria and several rodent *Plasmodium* species are now in widespread use, including *Plasmodium berghei*, *Plasmodium chabaudi*, *Plasmodium yoelii*, and *Plasmodium vinckei*. These species display substantial genotypic and phenotypic similarities to the human malarias *Plasmodium falciparum* and *Plasmodium vivax*[26] and, therefore, offer the potential to explore invasion phenotypes *in vivo* in mice. However, to use these models two issues need to be addressed.

Firstly, it is often challenging to accurately determine parasitaemia in *in vivo* samples. This is particularly pertinent at low parasitaemia levels when microscopic examination of blood smears is impractical. Automated methods such as flow cytometry are preferred, but accuracy can be hindered by the presence of additional cell types, especially RBC progenitors and leukocytes. Recently, several studies have reported the use of novel dyes combined with autofluorescence or fluorescently conjugated antibodies to accurately determine parasitaemia *in vivo*[27-29] and these are explored in this study. Another option is to use transgenic green fluorescent protein (GFP) expressing rodent malarial parasites as described by Franke-Fayard *et al.*[30]. However, the use of these parasites is so far restricted to two species of rodent malaria, *P. berghei* strain ANKA [30] and *P. yoelii*[31], with other parasite strains less suitable for transgenesis due to difficulties in maintaining the parasite *in vitro*. Another limitation of these parasites is that they must be maintained under constant drug selection to preserve a pure GFP expressing line. The second issue preventing accurate determination of invasion efficiency *in vivo* is the variation in synchronicity and parasitaemia between individual animals, which is not as pertinent a problem when using *in vitro* cultures. This variation is due to factors inherent in *in vivo* studies such as imperfect injection of the starting dose of parasites, small differences in the age or weight of individual animals, parasite variability and natural variation in the immune response to infection. Furthermore, during an *in vivo* infection, invasion can occur over a period of six hours or more making it difficult to distinguish between invasion and early stage growth phenotypes. To overcome this drawback it is necessary to discriminate between newly invaded parasitized RBCs (pRBCs) and those pRBCs already in circulation. This can be achieved by fluorescently labelling RBCs before exposing them to the parasite as previously described for *in vitro* assays [17,18]. Additionally, in order to avoid inaccuracies due to inter-individual variation it is also necessary to include a second population of labelled cells to act as a control. In this way the treated RBCs, or RBCs of interest, can be compared to control RBCs within one animal, thereby negating variations in parasite synchronicity and environmental conditions.

The study presented here describes a novel flow cytometric *in vivo* invasion assay, which addresses these issues. The assay was developed and optimized using mice infected with *P. chabaudi adami* strain DS*,* and its ability to analyse treatments known to block invasion in *in vitro* studies was verified. The assay allowed accurate determination of *in vivo* parasitaemia in mice, and distinguishes leukocytes, RBC progenitors, and RBCs containing Howell Jolly bodies. The ability of this assay to analyse the precise time of parasite invasion and correct for inter-individual variability through the use of two distinct RBC labels was also demonstrated.

## Methods

### Mice and parasites

Mice were housed under controlled temperature (21°C) with a 12:12 hr light-dark cycle. All procedures were conducted in accordance with the policies of Macquarie University and conformed to the National Health and Medical Research Council (NHMRC) Australian code of practice. The work was performed under the agreement Ethics No ARA 2012/017 approved and obtained from the Animal Ethics Committee at Macquarie University.

For experimental malaria infection stock blood infected with *P. chabaudi adami* DS or *P. berghei* ANKA was stored at -80°C. 250 μL of thawed parasitized blood was injected into the intraperitoneal cavity of C57BL/6 donor mice. Once C57BL/6 donors reached 5-15% parasitaemia they were anesthetized with isofluorane and bled by cardiac puncture before being sacrificed. Parasitized blood was diluted in Krebs’s buffered saline containing 0.2% glucose according to Jarra and Brown [32], and 1 × 10^4^ parasitized RBCs were injected into the intraperitoneal cavity of mice to be infected. All experiments were performed on SJL/J mice unless otherwise stated. Mice were monitored daily by tail bleed using microscopy or flow cytometry as described.

### Microscopy

Microscopy was used to determine parasitaemia of thin blood smears or to analyse cells sorted by flow cytometry. Sorted cells were spun down and concentrated before being allowed to settle onto glass slides coated with 0.1% polyethylenimine (PEI) (Sigma-Aldrich, St Louis, MO). Cells were fixed in methanol for one minute before being stained in a 10% Giemsa solution (Sigma-Aldrich, St Louis, MO) at pH 7.4 for 10mins. Parasitaemia was calculated by counting at least 500 parasitized cells by light microscopy at 100 × magnification.

### Staining of blood samples

Blood samples were prepared for flow cytometry using the following protocol. 3 μL of tail blood was collected directly into 50 μL staining solution which contained 12 μM JC-1 (Life Technologies, Carlsbad, CA), 5 μM Hoechst 33342 or 2 μM Hoechst 34580 (Sigma-Aldrich, St Louis, MO), 1 μg/mL Streptavidin PE-Cy7, 1 μg/mL anti-CD45 APC eFluor 780 (clone 30-F11), and 1 μg/mL anti-CD71 PerCP eFluor 710 (clone R17217) (eBioscience San Diego, CA) in MT-Ringer Complete (154 mM NaCL, 5.6 mM KCl, 1 mM MgCl2, 2.2 mM CaCl2, 20 mM HEPES, 10 mM glucose, 0.5% BSA, 30 U/mL heparin, pH 7.4, 0.22 μM filter sterilized) pre-warmed to 37°C. Samples were incubated at 37°C for 20 mins before adding 650 μL ice cold MT-Ringer Complete. Cells were then centrifuged at 750 g for 3 mins at 4°C and re-suspended in 700 μL MT-Ringer Complete before being analysed using flow cytometry. Under excitation at 488 nm, JC-1 exhibits a maximum emission at 530 nm, but at high concentrations it forms aggregates, which shift the emission to 580 nm. In this author’s experience JC-1 stained pRBCs produced very little fluorescence at 580 nm, therefore, only results obtained at 530 nm fluorescence are reported. For the evaluation of SYTO-16, Dihydroethidium, and Thiazole orange uninfected samples were prepared as described [27,29,33]. Briefly, cells were incubated with 1 μg/mL anti-CD45 APC eFluor 780 and 1 μg/mL anti-CD71 PerCP eFluor 710, along with either 2.5 μM SYTO-16, 5 μg/mL Dihydroethidium, or 100 ng/mL Thiazole Orange for 20 mins at room temperature.

### Determining the sensitivity of Hoechst 33342 and JC-1 detection of pRBCs

To assess the sensitivity of the Hoechst/JC-1 staining method at a different parasitaemia, blood from an infected mouse at approximately 1% parasitaemia was serially diluted with uninfected blood. Mice were anesthetized with isofluorane and bled by cardiac puncture before being sacrificed. Infected blood was divided into three aliquots and each aliquot was serially diluted threefold with uninfected blood a total of five times. The expected parasitaemia was calculated as the initial parasitaemia (determined by counting of parasites on a Giemsa stained slide) divided by the dilution factor. For each dilution the parasitaemia was measured using the Hoechst/JC-1 flow cytometry method described above as well as by using Hoechst 33342 or JC-1 fluorescence alone.

### Enzymatic treatment of RBCs

In order to validate the ability of this method to detect the inhibition of parasite invasion, RBCs were treated with enzymes reported to inhibit *P. falciparum* invasion *in vitro* as previously described [17]. Briefly, RBCs were incubated with 20 mU/mL neuraminidase, 0.5 mg/mL trypsin, and 1 mg/mL chymotrypsin in MT-Ringer Complete for 30 mins at 37°C.

### Labelling of RBCs

Donor blood was labelled and transfused into infected mice using the following protocol. Blood was collected by cardiac puncture of anesthetized mice and immediately combined with a one tenth volume of 10× heparin solution (300 U/mL heparin). Blood was kept at 4°C at all times. RBCs were treated as described or left untreated before labelling. For RBC labelling cells were suspended at 20% haematocrit in MT-Ringer (154 mM NaCL, 5.6 mM KCl, 1 mM MgCl2, 2.2 mM CaCl2, 20 mM HEPES, pH 7.4, 0.22 uM filter sterilized) with either 10 μg/mL of Hydroxysulfosuccinimide Atto 633 (NHS-Atto 633) (Sigma-Aldrich, St Louis, MO) or 125 μg/mL Sulfosuccinimidyl-6-(biotinamido)hexanoate (Sulfo-LC-NHS-Biotin) (Thermo Fisher Scientific, Waltham, MA) and incubated at 4°C for 1 hr with constant slow mixing. Stock solutions of 2 mg/mL NHS-Atto 633 and 25 mg/mL Sulfo-LC-NHS-Biotin were prepared in dimethylformamide (DMF) and stored at -20°C. Labelled RBCs were washed three times with MT-Ringer and resuspended in MT-Ringer at 40% haematocrit. Treated and untreated blood was combined in approximately equal proportion in the two label combinations (untreated Atto633/treated Biotin, untreated Biotin/treated Atto633). 200 μL of solution (approximately 2 × 10^9^ RBCs) was injected intravascularly into mice at 2-5% parasitaemia during peak schizogony and blood samples were taken 30 mins and 3 hrs after injection. Schizogony was at its peak approximately 6 hrs into the dark cycle.

### Flow cytometry and cell sorting

Samples were run and 1,000,000-10,000,000 events were collected using either BD FACS Diva or BD FACS Sortware with a BD Aria II or BD Influx cell sorter respectively (BD Biosciences, Franklin Lakes, NJ). The BD Aria II was equipped with a 50 mW 405 nm laser, 20 mW 488 nm laser, and 18 mW 633 nm laser while the BD Influx was equipped with a 100 mW 355 nm laser, 200 mW 488 nm laser, and 120 mW 640 nm laser. Hoechst 33342 was excited using the 355 nm laser and detected through a 460/50 filter. Hoechst 34580 was excited using the 405 nm laser and detected through a 460/50 filter. JC-1, anti-CD71 PerCP eFluor 710 and Streptavidin PE-Cy7 were excited using the 488 nm laser and detected through 530/40, 692/40, and 750LP filters respectively. Atto633 and anti-CD45 APC eFluor 780 were excited using the 633 nm laser and detected through 670/30, 750LP filters respectively. The RBC and leukocyte population was selected based on FSC/SSC properties and single cells were gated based on either trigger pulse width or by using the FSC peak area to height ratio. Cell sorting was performed on the BD Influx into collection tubes and slides prepared as described earlier. Compensation and further analysis was performed using FlowJo v10.0.6 (Tree Star, Ashland, Oregon, USA).

## Results

### A novel flow cytometric method to detect parasitized RBCs in *in vivo* samples

**Figure 1:**
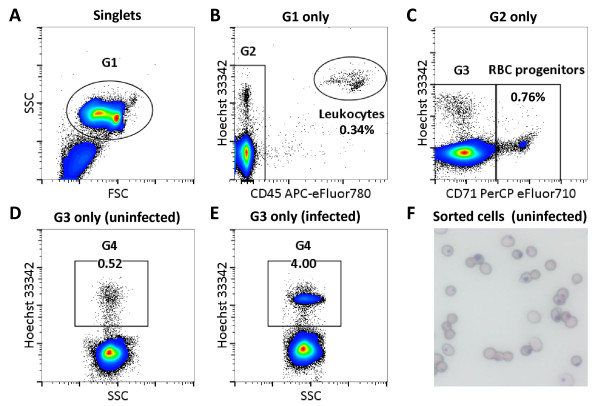
**Gating strategy of uninfected whole blood from SJL/J mice.** Single cells were gated based on trigger pulse width and from these cells, debris, noise and platelets were gated out based on forward scatter/side scatter (FSC/SSC) as G1 **(A)**. Leukocytes were then gated out by selecting anti-CD45 APC eFluor780 negative cells (G2) **(B)** and RBC progenitor cells were gated out by selecting anti-CD71 PerCP eFluor710 negative cells (G3) **(C)**. Finally the remaining cells were gated based on positive DNA staining by Hoechst 33342 (G4). Representative results are shown from uninfected **(D)** and *P. chabaudi adami* DS infected **(E)** mice. At each stage only the cells in the previous gate were analysed as indicated. A representative image of cells from gate G4 in uninfected mice, which were sorted and Giemsa stained is shown, these are characteristic Howell-Jolly bodies **(F)**.

In preliminary studies using uninfected mice, a flow cytometric cell staining and analysis protocol was tested that took advantage of the DNA-specific dye, Hoechst 33342, which distinguishes DNA-containing blood cells [33], in conjunction with fluorescently-labelled antibodies raised against the leukocyte common antigen (CD45) and a red cell progenitor marker (CD71) to exclude leukocytes and non-parasitized nucleated blood cells. It was found that even after excluding Hoechst-CD45 and Hoechst–CD71 dual-positive events, there remained a Hoechst-stained population accounting for 0.3-0.9% of blood cells (Figure 1A-D). Furthermore, the Hoechst fluorescence intensity of these cells was equal to that of pRBCs (Figure 1E). To determine their identity, the cells were sorted onto glass slides, fixed, stained with Giemsa and examined under light microscopy. Most of the cells resembled red blood cells, with the addition of intracellular spherical basophilic-stained particles consistent with the appearance of Howell-Jolly (HJ) bodies (Figure 1F). HJ-RBCs were observed at similar frequencies in both SJL/J and C57BL/6 strains of mice, and were also detected when other nucleic acid-specific dyes, including SYTO-16, Dihydroethidium, and Thiazole Orange were used (Additional file 1).

**Figure 2:**
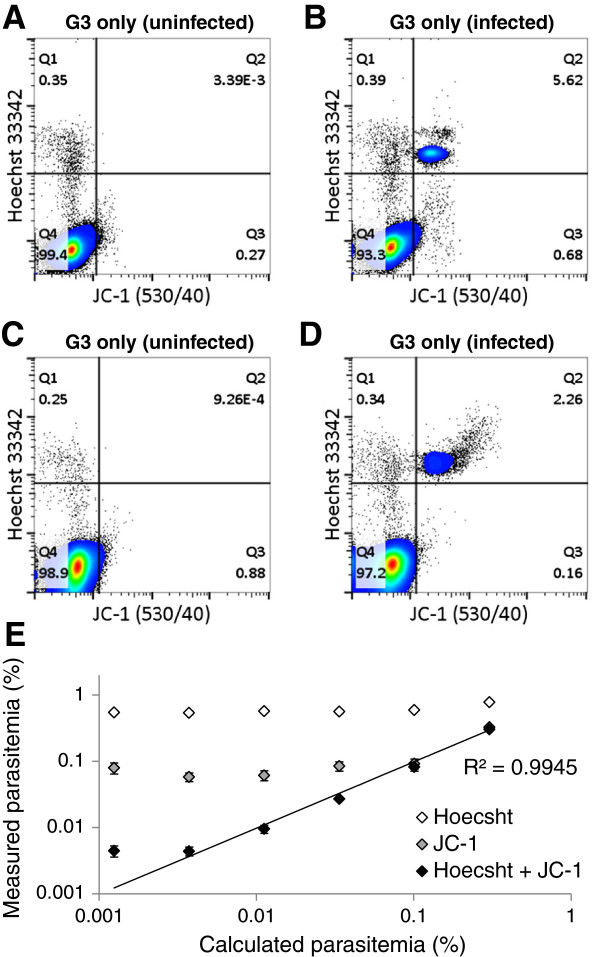
**Hoechst and JC-1 staining of uninfected and infected blood.** In one experiment blood samples were collected from uninfected **(A)** and *P. chabaudi adami* DS infected **(B)** mice. In another experiment blood was collected from uninfected **(C)** and *P. berghei* ANKA infected **(D)** mice. Blood samples were stained with JC-1, anti-CD45 APC eFluor780, anti-CD71 PerCP eFluor710, and Hoechst 33342. Note that plots are a typical representation of results and instrument voltages were slightly different between experiments. Samples were gated up to G3 as in Figure 1. A blood sample from a *P. chabaudi adami* DS infected mouse at approximately 1% parasitaemia was split into three aliquots and serially diluted with uninfected blood to create a dilution curve **(E)**. Calculated parasitaemia was estimated based on the parasitaemia of the undiluted sample, determined by light microscopy, and the dilution factor. Measured parasitaemia for Hoechst was calculated as Q1 + Q2, for JC-1 it was calculated as Q2 + Q3 and for Hoechst + JC-1 it was calculated as Q2 alone **(D)**. Error bars represent SEM for the three replicates.

To distinguish HJ-RBCs from pRBCs in blood from infected mice, the inclusion of a mitochondrial membrane potential dye, JC-1, which has been used previously in *P. falciparum* studies to determine parasite viability, was investigated [34]. In uninfected samples, fewer than 0.005% of cells were Hoechst 33342 and JC-1 positive when leukocytes and RBC progenitors were excluded from analyses (Figure 2A). That is, the vast majority of Hoechst positive red cells identified above as containing HJ-bodies, did not stain with JC-1. To determine the optimal staining conditions of JC-1 blood samples from *P. chabaudi adami* DS infected mice at 2-10% parasitaemia were prepared. Concentrations ranging from 0.75 μM to 24 μM were tested and it was found that optimal staining conditions were incubation with 12 μM of JC-1 for 20 mins at 37°C (Additional file 2). Under these staining conditions infected samples contained a population of Hoechst 33342 positive and JC-1 positive cells, which corresponded to sample parasitaemia (Figure 2B). Samples from *P. berghei* ANKA infected mice exhibited a similar staining pattern indicating this method is consistent across different parasite species (Figure 2C, D). In order to make this technique more broadly accessible, another DNA dye, Hoechst 34580, was tested, which can be used with a standard 405 nm violet laser. Results indicate that although Hoechst 34580 results in less separation between uninfected and infected RBC populations, it can be used effectively when combined with JC-1 and provides similar sensitivity to Hoechst 33342 (Additional file 3). To test the sensitivity of this staining protocol in distinguishing parasite-infected cells, infected blood was serially diluted with uninfected blood, and the percentage of infected cells determined (% parasitaemia). The limit of detection was approximately 0.007% when using JC-1 and Hoechst 33342 together compared to 0.64% when using Hoechst 33342 alone (Figure 2E). Finally, this protocol was assessed in the absence of anti-CD71 and anti-CD45 antibodies. Although sensitivity was reduced due to an inability to distinguish leukocytes from infected cells, this method reduced costs and simplified sample analysis (Additional file 4).

### *In vivo* parasite invasion assay

**Figure 3:**
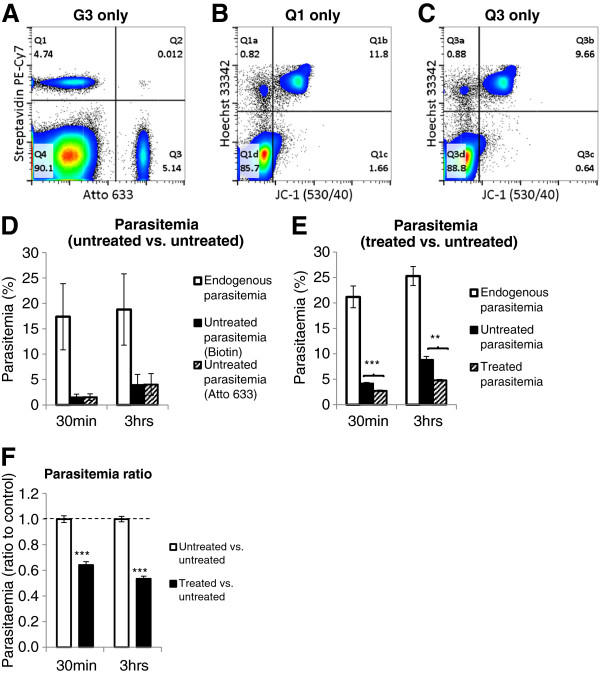
***In vivo*parasite invasion assay.** Blood samples were taken from *P. chabaudi adami* DS infected SJL/J mice 30 mins and 3 hrs after injection with labelled RBCs and stained with JC-1, Hoechst 33342, anti-CD45 APC eFluor780, anti-CD71 PerCP eFluor710 and Streptavidin PE-Cy7. Cells were gated to G3 as described in Figure 1 and then gated based on their respective label **(A)**. Cells from Q1 (Biotin labelled) were gated based on JC-1 and Hoechst fluorescence and the parasitaemia was determined as Q1b divided by Q1 **(B)**. Similarly, parasitaemia of cells from Q3 (Atto 633 labelled) was determined as Q3b divided by Q3 **(C)**, and endogenous parasitaemia was determined based on the cells from Q4 (unlabelled). As a control experiment the two labelled RBC populations were untreated **(D)**. To quantify the effect of protease treatment on invasion one labelled RBC population was treated with trypsin, chymotrypsin and neuraminidase, while the other was left untreated **(E)**. Parasitaemia ratio calculated as the parasitaemia of the labelled RBCs of interest divided by the parasitaemia of the labelled control RBCs for each individual sample **(F)**. Results are from five or six infected mice with RBC labels switched between treated and untreated to account for any invasion difference due to the RBC label. Error bars represent SEM, **p-value < 0.01, ***p-value < 0.001.

In addition to a method for quantifying parasitaemia, a flow cytometric method for quantifying parasite invasion *in vivo* was developed. A major hurdle in this type of analysis is distinguishing parasite invasion from parasite growth or clearance. To do this it is necessary to distinguish newly infected RBCs from those already in circulation. This was achieved by labelling two populations of RBCs and transfusing them into infected mice. Two criteria had to be met in selecting suitable RBC labels for this assay. Firstly, labels had to be compatible with the Hoechst/JC-1 method of pRBC detection and secondly, allow parasite invasion to occur as normal. Hydroxysulfosuccinimide Atto 633 (NHS-Atto 633) and sulfosuccinimidyl-6-(biotinamido) hexanoate (Sulfo-LC-NHS-Biotin) were selected as suitable labels. The NHS conjugate binds to primary amines on the surface of RBCs. Both labels were optimized for the minimum concentration required to distinguish labelled cells from unlabelled, and were clearly distinguishable from each other without need for compensation (Figure 3A).

To determine that the RBC labels did not affect the ability of parasites to invade RBCs, blood conjugated with the two different labels was combined and injected into infected SJL/J mice during schizogony at 2-10% parasitaemia. The time of day was critical in this assay in order to maximize the number of invasion events. It was determined that schizogony peaked approximately half way through the dark cycle, under the conditions used here, this corresponded to between 10 pm and 2 am. Blood was sampled 30 mins and 3 hrs after injection, and stained and analysed as described in Methods. The results showed that the parasitaemia of the two labelled populations for each of the infected mice were virtually identical (Figure 3D, F and Additional file 5), indicating that these labels do not differentially affect parasite invasion. It was however observed that the parasitaemia of the fluorescently labelled populations varied between mice and did not correlate with the endogenous parasitaemia of the host mouse (Additional file 5). This was presumably due to variations in the number parasites undergoing schizogony in each mouse during the course of the assay.

It was next determined if this method could be used to compare rates of invasion between normal cells, and cells treated *ex vivo* with a combination of proteases (neuraminidase, trypsin, and chymotrypsin) that are known to remove host cell molecules necessary for merozoite interaction and entry into red cells [5,8]. Blood collected from a donor mouse was divided in two. One aliquot was subjected to protease treatment, and the other was left untreated. Aliquots were labelled, combined and injected into infected mice during peak schizogony (see Additional file 6 for schematic). Blood samples were collected at 30 mins and 3 hrs after injection and parasitaemia of the two populations determined (Figure 3B, C, and E). Similar to the above-mentioned assay results, parasitaemia values varied between individual mice (Additional file 5). However, at each time point the parasitaemia of protease-treated cells was 30-60% less than in untreated cells in the same mouse, corresponding to parasitaemia ratios ranging between 0.4 and 0.7. These differences were observed at each time point, were irrespective of the dye combination, and were highly statistically significant (Figure 3F and Additional file 5).

## Discussion

In this report, a novel flow cytometry based assay is presented which allows the quantification of erythrocytic parasite invasion *in vivo.* To develop this assay the specificity of current fluorescent dyes used for the detection of pRBCs were evaluated in an *in vivo* model of malarial infection. To do this, several DNA specific dyes alone or in combination previously reported for parasitaemia measurement were assessed: Hoechst 33342 [33,35], SYTO 16 [27], Dihydroethidium [29,36], and Thiazole Orange [33,37]. Surprisingly, it was found that samples from uninfected mice stained with these dyes resulted in a positively stained population of 0.3-0.9% which was considerably larger than previously reported values for *in vivo* analysis [27-29]. To explain this discrepancy, this cell population was isolated and examined. This resulted in the detection of basophilic intra-erythrocytic staining indicating the presence of Howell-Jolly (HJ) bodies. HJ-RBCs are usually quite rare in humans, and are associated with abnormal splenic function [38]. In mice, some studies report HJ-RBC frequencies in control animals comparable to the data obtained in this study [39,40] while others report lower levels [41], it is not clear why this is the case. HJ-RBCs occur when remnants of DNA remain in mature RBCs due to incomplete expulsion of the nucleus during erythropoiesis. As pRBCs and HJ-RBCs could not be distinguished from each other based on DNA staining alone, JC-1, a mitochondrial membrane dye, was investigated to determine if it would allow for increased specificity in pRBC staining. It was found that the combination of mitochondrial (JC-1) and nucleic acid stain (Hoechst 33342) provided an increase in the LOD of pRBCs from 0.64% to 0.007% compared with Hoechst 33342 alone. Similar levels of sensitivity were observed in mice infected with *P. chabaudi adami* DS or *P. berghei* ANKA. Although mitochondrial membrane potential dyes have previously been employed to assess parasite viability [34,42] and to determine parasitaemia [43,44] to the best of this author’s knowledge the combination of these with DNA specific dyes has not been used to quantify parasitaemia *in vivo*. In addition to JC-1 and Hoechst, selective, fluorescently labelled antibodies were employed to detect and exclude RBC progenitors and leukocytes, further improving the sensitivity of the assay. As well as using the Hoechst 33342 dye, which must be excited with a 355 nm (UV) laser, the use of an alternative Hoechst 34580 dye, which is excited by the more commonly available 405 nm laser, was demonstrated, offering a broader applicability for this assay. The later dye has been used previously to measure parasitaemia [45]. In a practical setting this assay allows accurate quantification of parasitaemia down to approximately 0.013% utilizing a 355/488/633 nm three-laser instrument, with the detection of pRBCs as low as 0.007%. However, in order to accurately measure low parasitaemia a sufficient number of events must be analysed to overcome the incidence of noise related to sample or machine impurities, in some cases this will require the analysis of > 5,000,000 events.

In addition to parasitaemia measurement a method was established to directly compare rates of parasite invasion in different RBC donor cells within a single recipient animal. RBC labels were evaluated that could be detected in conjunction with the Hoechst and JC-1 dyes, and the NHS-Atto 633 and Sulfo-LC-NHS-Biotin (combined with streptavidin PE-Cy7) were selected. To ensure the accuracy of this assay the effect these labels might have on parasite invasion was addressed. Theron *et al.*[17] suggested that surface labels, such as FITC, may inhibit invasion, while Pattanapanyasat *et al.*[46] report that using biotin as a surface label has no effect on invasion. Under the conditions used here, labelling red cells with these molecules did not affect parasite invasion *in vivo*. The high quantum yield of the molecules allowed concentrations of the labels to be minimized. By using two populations of labelled cells, rather than one population as used in *in vitro* assays [17,18,46], the assay was able to be performed in mice with variable parasite loads and parasite stage synchronicity with little effect on results. In addition, by optimizing the starting time of this assay to coincide with peak schizogony significant numbers of newly invaded RBCs were detected after just 30 minutes; this timeframe was also sufficient to detect differential invasion rates between protease-treated and untreated cells. The limited time frame is likely to specifically reflect an invasion phenotype, rather than parasite growth as reported in previous assays [45]. However, results suggest that by continuing this assay over longer time periods other aspects of the parasite life cycle such as growth, splenic clearance, and sequestration can also be investigated.

Once this assay was established it was determined if invasion inhibition produced by treatment of RBCs with trypsin, chymotrypsin, and neuraminidase could be detected. It was found that protease treatment reduced invasion by 35%. This effect on invasion was not as great as what may be expected, although treatments such as this have been shown to have variable effect between different strains of *P. falciparum* parasites [18]. Importantly, the magnitude of the invasion inhibition was highly consistent between mice despite differences in parasitaemia and synchronization, and was not affected by label combination.

## Conclusions

The ability to accurately study the interaction between the parasite and its host cell is of utmost importance in determining factors which are essential for parasite survival. To date, techniques for assessing parasite invasion have been exclusively carried out *in vitro*. The assay presented here allows the accurate measurement of both parasitaemia and erythrocytic invasion *in vivo.* The validity of this assay to detect invasion inhibition was demonstrated.

## Competing interests

The authors declare that they have no competing interests.

## Authors’ contributions

PML wrote the manuscript, helped conceive the study, and carried out all experiments with the exception of maintaining the parasite lines and performing mouse malaria infections; these were carried out by SL and GB. BJM, SJF, and GB helped conceive the study, contributed toward experimental design and analysis, and assisted in drafting the manuscript. All authors read and approved the final manuscript.

## Supplementary Material

Additional file 1 **Background staining using nucleic acid-specific dyes.** Background staining of HJ-RBCs in uninfected mice was observed when using the nucleic acid-specific dyes SYTO 16 (A), Thiazole orange (C) and Dihydroethidium (E). These populations were indistinguishable from parasitized cells in equivalent samples from infected mice (B, D, and F). Click here for file

Additional file 2 **Optimization of JC-1 staining.** A blood sample was taken from a *P. chabaudi adami* DS infected mouse and incubated with different concentrations of JC-1. Cells were analysed by flow cytometry (A), and the optimal staining concentration was 12uM as determined by the ratio of the mean fluorescence intensity (MFI) of the JC-1 positive population compared to the negative population (B). Click here for file

Additional file 3 **Hoechst 34580 and JC-1 staining of uninfected and infected blood.** Blood samples were collected from uninfected (A) and *P. chabaudi adami* DS infected (B) mice and stained with JC-1, anti-CD45 APC eFluor780, anti-CD71 PerCP eFluor710, and Hoechst 34580. Samples were gated up to G4 as in Figure 1 except that the forward scatter peak height to area ratio was used to distinguish single cells rather than trigger pulse width. Click here for file

Additional file 4 **Hoechst and JC-1 staining of uninfected and infected blood without using antibodies.** Blood samples were collected from uninfected (A) and *P. chabaudi adami DS* infected (B) mice and stained as in Figure 2. Samples were gated based on trigger pulse width and FSC/SSC up to G1 as in Figure 1 without using antibody staining to remove leukocytes and reticulocytes from the analysis. The overlap between mature red blood cells (red), leukocytes (orange) and reticulocytes (blue) is shown (C, D). Click here for file

Additional file 5 **Results from individual infected mice included in the*in vivo*parasite invasion assay.** Complete data set for each mouse from the *in vivo* parasite invasion assays. Click here for file

Additional file 6 **Schematic representation of the*In vivo*parasite invasion assay.** Blood was collected from uninfected SJL/J mice and divided into two tubes. One tube is treated with neuraminidase, trypsin, and chymotrypsin, which are known to inhibit parasite invasion while the other sample is left untreated (A). These tubes are again divided into two tubes and one is labelled with Biotin-NHS and the other with Atto 633-NHS (B). Samples were then combined in two combinations; Biotin labelled treated RBCs with Atto 633 labelled untreated RBCs and Atto 633 labelled treated RBCs with Biotin labelled untreated RBCs (C). These two combinations were injected separately into two lots of infected mice during schizogony at 2-10% parasitaemia (D). Click here for file
