# Supplementary material for: A flow cytometric assay to quantify invasion of red blood cells by rodent Plasmodium parasites in vivo
Source: Malar J. 2014 Mar 17;13:100. doi: 10.1186/1475-2875-13-100 (PMC4004390; doi:10.1186/1475-2875-13-100)
Supplement: Additional file 1 — Background staining using nucleic acid-specific dyes. Background staining of HJ-RBCs in uninfected mice was observed when using the nucleic acid-specific dyes SYTO 16 (A), Thiazole orange (C) and Dihydroethidium (E). These populations were indistinguishable from parasitized cells in equivalent samples from infected mice (B, D, and F). [file 1475-2875-13-100-S1.pdf]

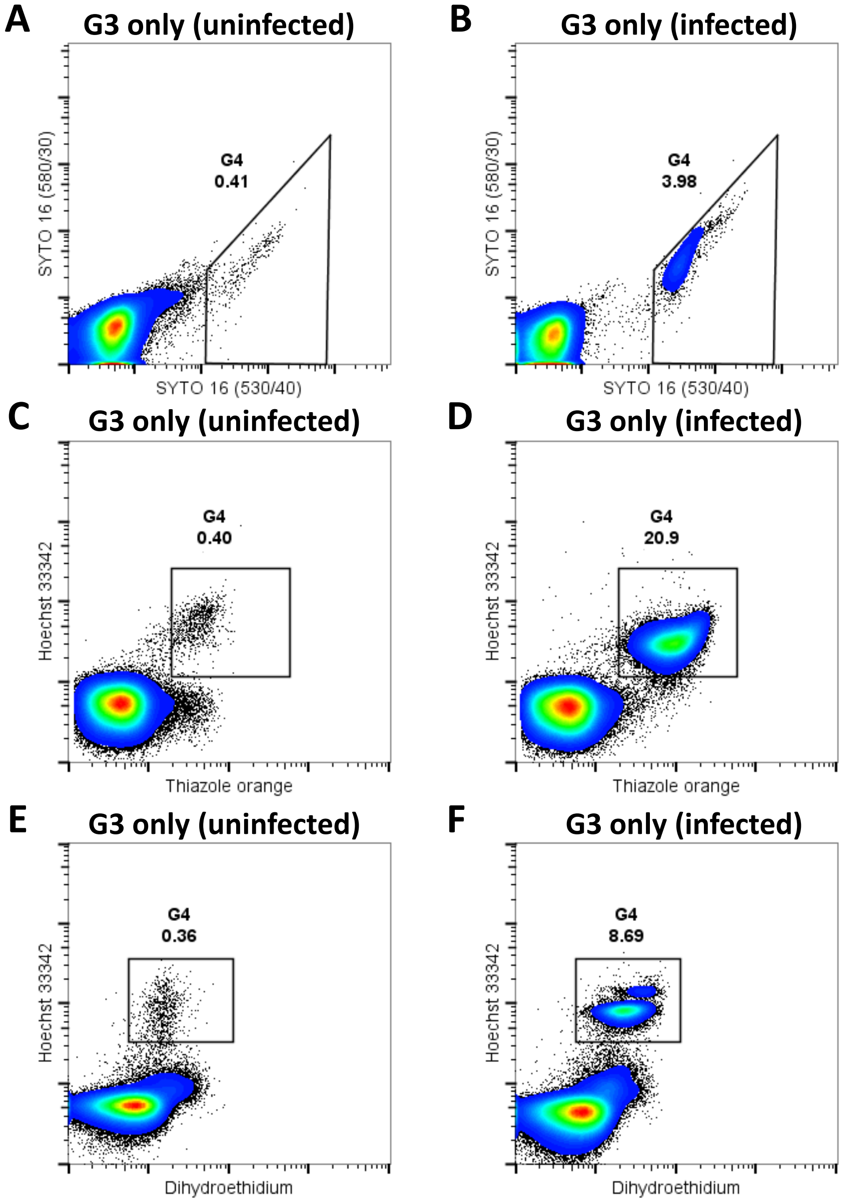

Additional file 1 – Background staining using nucleic acid-specific dyes

Background staining of HJ-RBCs in uninfected mice was observed when using the nucleic acid-specific dyes SYTO 16 (A), Thiazole orange (C) and Dihydroethidium (E). These populations were indistinguishable from parasitized cells in equivalent samples from infected mice (B, D, and F)
