# Supplementary material for: A flow cytometric assay to quantify invasion of red blood cells by rodent Plasmodium parasites in vivo
Source: Malar J. 2014 Mar 17;13:100. doi: 10.1186/1475-2875-13-100 (PMC4004390; doi:10.1186/1475-2875-13-100)
Supplement: Additional file 2 — Optimization of JC-1 staining. A blood sample was taken from a P. chabaudi adami DS infected mouse and incubated with different concentrations of JC-1. Cells were analysed by flow cytometry (A), and the optimal staining concentration was 12uM as determined by the ratio of the mean fluorescence intensity (MFI) of the JC-1 positive population compared to the negative population (B). [file 1475-2875-13-100-S2.pdf]

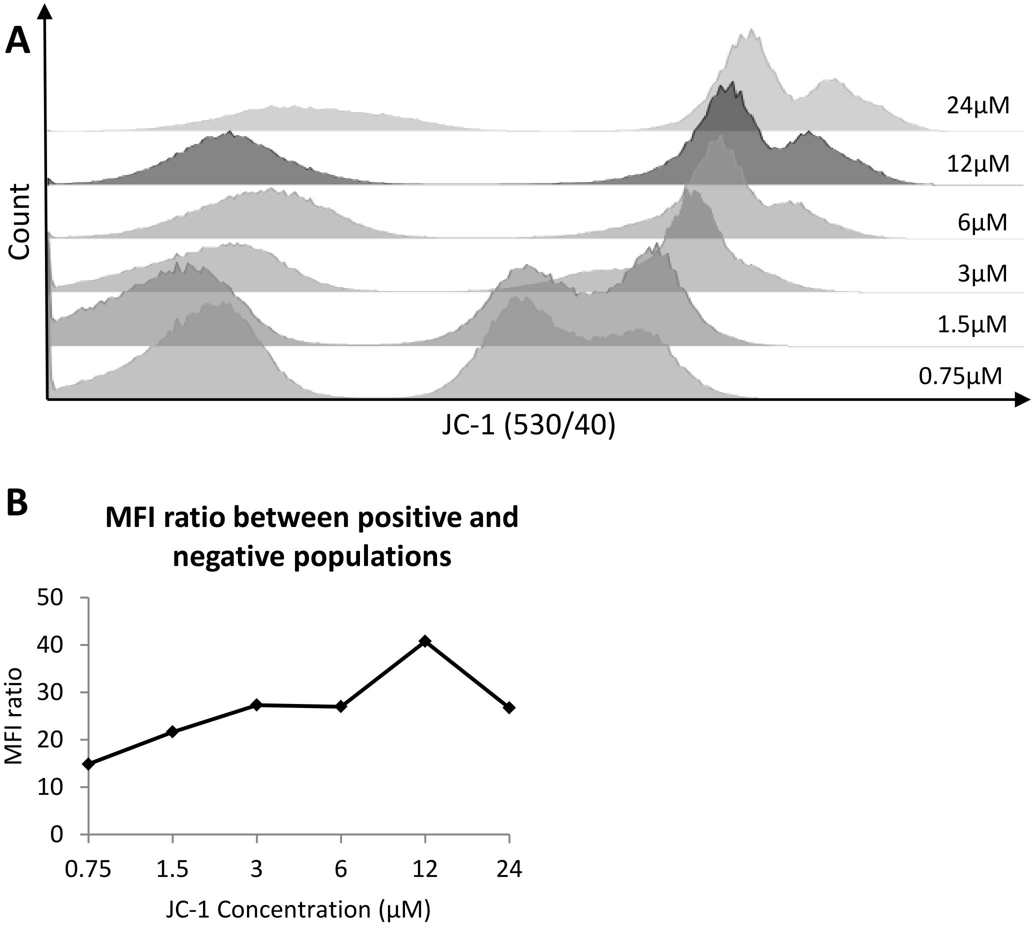

Additional file 2 - Optimization of JC-1 staining

A blood sample was taken from a *P. chabaudi adami DS* infected mouse and incubated with different concentrations of JC-1. Cells were analyzed by flow cytometry (A), and the optimal staining concentration was 12uM as determined by the ratio of the mean fluorescence intensity (MFI) of the JC-1 positive population compared to the negative population (B).
