# Supplementary material for: A flow cytometric assay to quantify invasion of red blood cells by rodent Plasmodium parasites in vivo
Source: Malar J. 2014 Mar 17;13:100. doi: 10.1186/1475-2875-13-100 (PMC4004390; doi:10.1186/1475-2875-13-100)
Supplement: Additional file 3 — Hoechst 34580 and JC-1 staining of uninfected and infected blood. Blood samples were collected from uninfected (A) and P. chabaudi adami DS infected (B) mice and stained with JC-1, anti-CD45 APC eFluor780, anti-CD71 PerCP eFluor710, and Hoechst 34580. Samples were gated up to G4 as in Figure 1 except that the forward scatter peak height to area ratio was used to distinguish single cells rather than trigger pulse width. [file 1475-2875-13-100-S3.pdf]

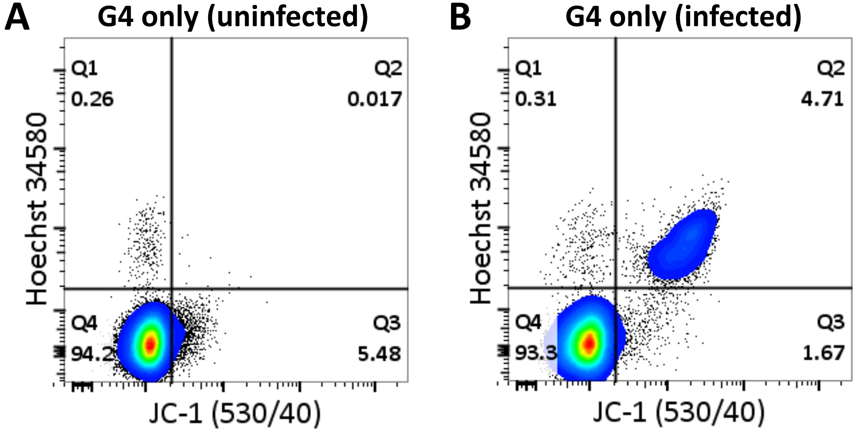

Additional file 3 - Hoechst 34580 and JC-1 staining of uninfected and infected blood

Blood samples were collected from uninfected (A) and *P. chabaudi adami DS* infected (B) mice and stained with JC-1, anti-CD45 APC eFluor780, anti-CD71 PerCP eFluor710, and Hoechst 34580. Samples were gated up to G4 as in Figure 1 except that the forward scatter peak height to area ratio was used to distinguish single cells rather than trigger pulse width.
