# Supplementary material for: A flow cytometric assay to quantify invasion of red blood cells by rodent Plasmodium parasites in vivo
Source: Malar J. 2014 Mar 17;13:100. doi: 10.1186/1475-2875-13-100 (PMC4004390; doi:10.1186/1475-2875-13-100)
Supplement: Additional file 4 — Hoechst and JC-1 staining of uninfected and infected blood without using antibodies. Blood samples were collected from uninfected (A) and P. chabaudi adami DS infected (B) mice and stained as in Figure 2. Samples were gated based on trigger pulse width and FSC/SSC up to G1 as in Figure 1 without using antibody staining to remove leukocytes and reticulocytes from the analysis. The overlap between mature red blood cells (red), leukocytes (orange) and reticulocytes (blue) is shown (C, D). [file 1475-2875-13-100-S4.pdf]

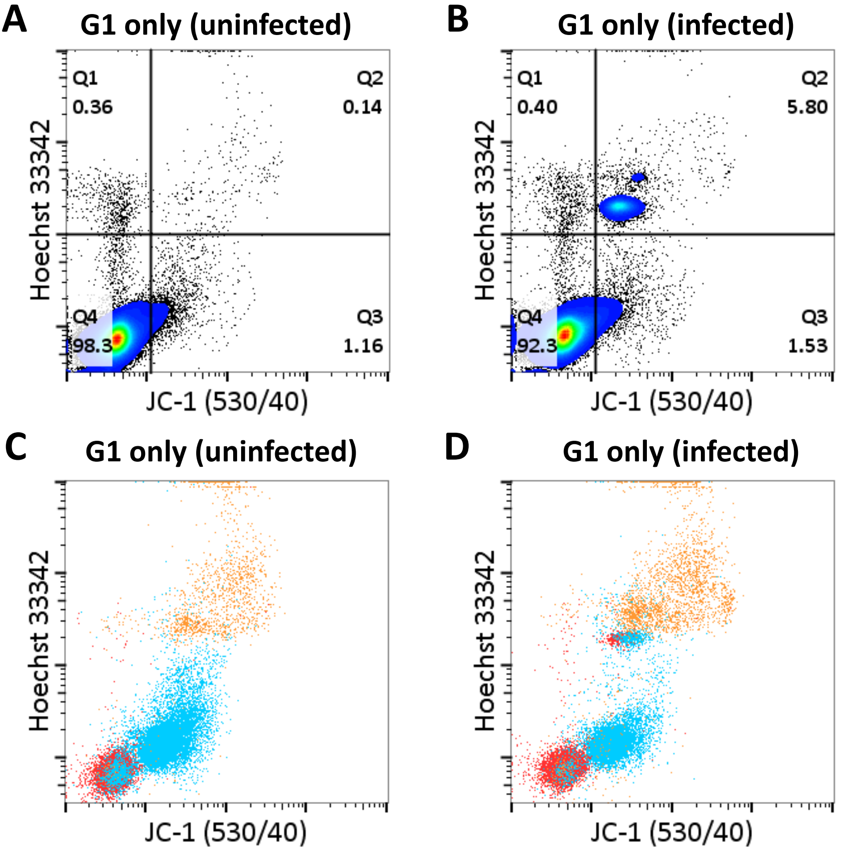

Additional file 4 –Hoechst and JC-1 staining of uninfected and infected blood without using antibodies

Blood samples were collected from uninfected (A) and *P. chabaudi adami DS* infected (B) mice and stained as in Figure 2. Samples were gated based on trigger pulse width and FSC/SSC up to G1 as in Figure 1 without using antibody staining to remove leukocytes and reticulocytes from the analysis. The overlap between mature red blood cells (red), leukocytes (orange) and reticulocytes (blue) is shown (C, D).
