# Supplementary material for: A flow cytometric assay to quantify invasion of red blood cells by rodent Plasmodium parasites in vivo
Source: Malar J. 2014 Mar 17;13:100. doi: 10.1186/1475-2875-13-100 (PMC4004390; doi:10.1186/1475-2875-13-100)
Supplement: Additional file 5 — Results from individual infected mice included in thein vivoparasite invasion assay. Complete data set for each mouse from the in vivo parasite invasion assays. [file 1475-2875-13-100-S5.pdf]

Table 1 - Results from individual infected mice included in the *in vivo* parasite invasion assay.

Mouse	Biotin label	Atto633 label	30 minutes after transfusion				3 hours after transfusion			
			Endogenous parasitemia*	Biotin parasitemia	Atto633 parasitemia	Parasitemia ratio#	Endogenous parasitemia*	Biotin parasitemia	Atto633 parasitemia	Parasitemia ratio#
**Untreated vs. untreated**										
**1**	Untreated	Untreated	8.20%	0.50%	0.50%	1.02	8.60%	0.80%	0.80%	1
**2**	Untreated	Untreated	25.10%	1.30%	1.30%	0.99	27.00%	3.80%	3.80%	1
**3**	Untreated	Untreated	42.40%	4.30%	4.40%	0.98	45.80%	12.90%	13.30%	0.97
**4**	Untreated	Untreated	2.80%	0.40%	0.40%	1	3.10%	0.50%	0.50%	0.94
**5**	Untreated	Untreated	8.30%	0.80%	0.90%	1.1	9.50%	1.70%	1.70%	1.03
**Treated vs. untreated**										
**1**	Treated	Untreated	30.00%	2.20%	4.10%	0.54	32.00%	4.50%	8.30%	0.54
**2**	Treated	Untreated	15.40%	3.10%	4.50%	0.68	20.00%	5.60%	10.00%	0.56
**3**	Treated	Untreated	15.80%	3.20%	4.90%	0.64	19.90%	4.80%	11.40%	0.42
**4**	Untreated	Treated	18.70%	4.30%	2.90%	0.68	23.40%	8.80%	5.20%	0.59
**5**	Untreated	Treated	21.50%	4.00%	2.70%	0.66	27.20%	8.20%	4.60%	0.56
**6**	Untreated	Treated	25.70%	3.20%	2.40%	0.76	29.20%	6.20%	4.10%	0.66

* Endogenous parasitemia is the parasitemia of unlabeled RBCs in each mouse.

Parasitemia ratio is treated parasitemia dived by untreated parasitemia
