# Supplementary material for: A flow cytometric assay to quantify invasion of red blood cells by rodent Plasmodium parasites in vivo
Source: Malar J. 2014 Mar 17;13:100. doi: 10.1186/1475-2875-13-100 (PMC4004390; doi:10.1186/1475-2875-13-100)
Supplement: Additional file 6 — Schematic representation of theIn vivoparasite invasion assay. Blood was collected from uninfected SJL/J mice and divided into two tubes. One tube is treated with neuraminidase, trypsin, and chymotrypsin, which are known to inhibit parasite invasion while the other sample is left untreated (A). These tubes are again divided into two tubes and one is labelled with Biotin-NHS and the other with Atto 633-NHS (B). Samples were then combined in two combinations; Biotin labelled treated RBCs with Atto 633 labelled untreated RBCs and Atto 633 labelled treated RBCs with Biotin labelled untreated RBCs (C). These two combinations were injected separately into two lots of infected mice during schizogony at 2-10% parasitaemia (D). [file 1475-2875-13-100-S6.pdf]

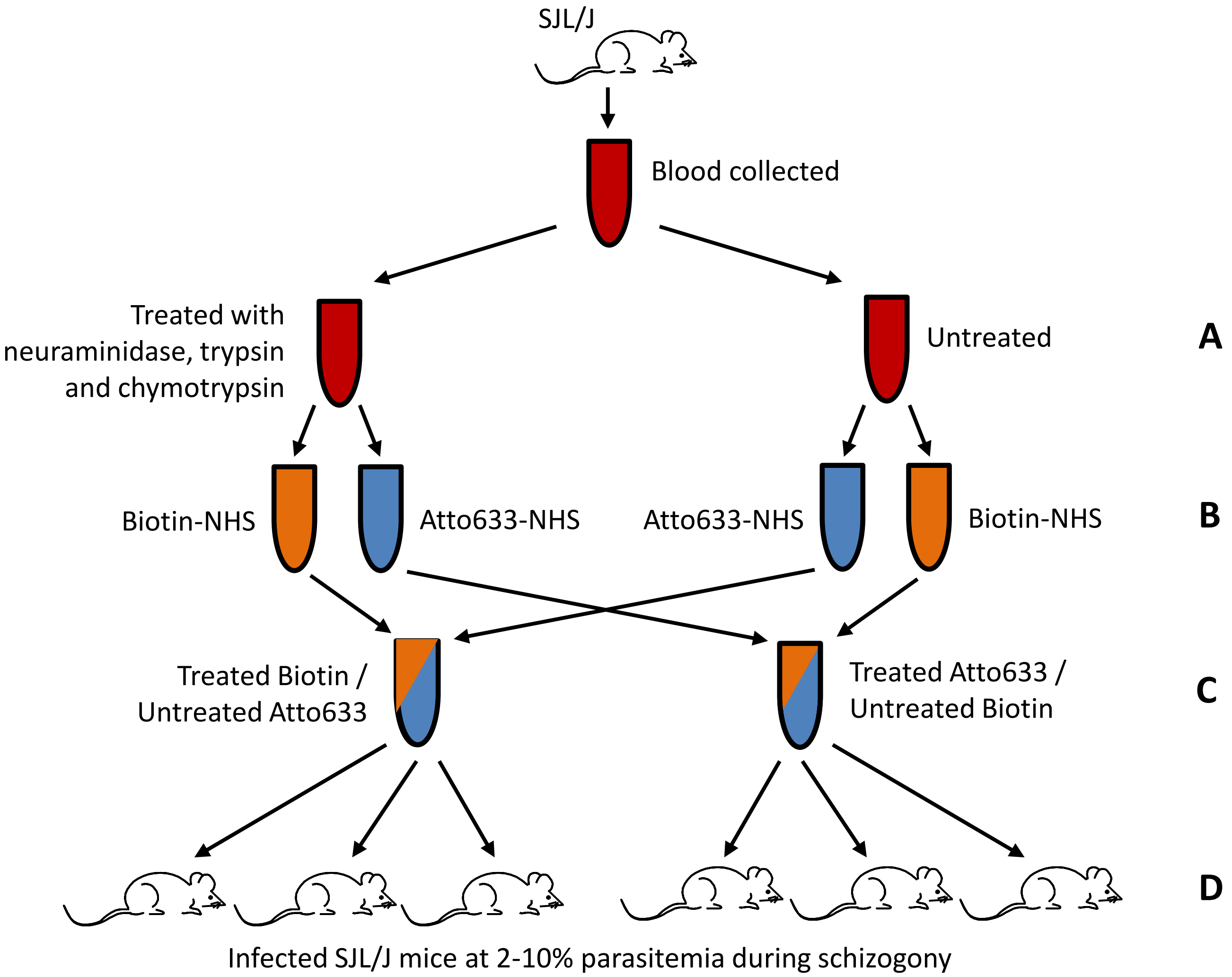

Additional file 5 - Schematic representation of the *In vivo* parasite invasion assay
Blood was collected from uninfected SJL/J mice and divided into two tubes. One tube is treated with neuraminidase, trypsin, and chymotrypsin, which are known to inhibit parasite invasion while the other sample is left untreated (A). These tubes are again divided into two tubes and one is labeled with Biotin-NHS and the other with Atto 633-NHS (B). We then combined the samples in two combinations; Biotin labeled treated RBCs with Atto 633 labeled untreated RBCs and Atto 633 labeled treated RBCs with Biotin labeled untreated RBCs (C). These two combinations were injected separately into two lots of infected mice during schizogony at 2-10% parasitemia (D).
